# Impact of Health Insurance on Health Care Utilisation and Out-of-Pocket Health Expenditure in Vietnam

**DOI:** 10.1155/2020/9065287

**Published:** 2020-08-26

**Authors:** Nguyen Thi Thu Thuong, Tran Quang Huy, Do Anh Tai, Tran Nhuan Kien

**Affiliations:** TNU-University of Economics and Business Administration, Vietnam Thai Nguyen 250000

## Abstract

**Background:**

In recent years, health insurance (HI) has been chosen by many low- and middle-income countries to obtain an important health policy target—universal health coverage. Vietnam has recently introduced the Revised Health Insurance Law, and the effects of the voluntary health insurance (VHI) and heavily subsidised health insurance (HSHI) programmes have not yet been analysed. Therefore, this study is aimed at examining the impact of these HI programmes on the utilisation of health care services and out-of-pocket health expenditure (OOP) in general and across different health care providers in particular.

**Methods:**

Using the two waves of Vietnam Household Living Standard Surveys 2014 and 2016 and the difference-in-difference method, the impacts of VHI and HSHI on health care utilisation and OOP in Vietnam were estimated.

**Results:**

For both the VHI and HSHI groups, we found that HI increased the probability of seeking outpatient care, the mean number of outpatient visits, the total number of visits, and the mean number of visits at the district level of health care providers in the last 12 months. However, there was no evidence that the HSHI programmes increased the mean number of inpatient visits and the number of visits at the provincial hospital. We also found that while the VHI programme reduced OOP for both outpatient and inpatient care, the HSHI scheme did not result in a reduction in OOP for hospitalisation, although HI lowered the total OOP. Similarly, we found that for both groups, HI reduced OOP when the insured visited district and provincial hospitals. However, the statistically significant impact was not demonstrated when the enrolees of HSHI programmes visited provincial hospitals.

**Conclusion:**

The study offers evidence that the Vietnamese HI scheme increased health care service utilisation and decreased OOP for the participants of the VHI and HSHI programmes. Therefore, the government should continue to consider improving the HI system as a strategy to achieve universal health coverage.

## 1. Introduction

Universal health coverage (UHC), which means that everyone can access sufficient quality health care services, including promotive, preventive, curative, rehabilitative, and palliative services without any financial difficulties, is a widely used concept, especially in low- and middle-income countries (LMIC) [1–5]. The World Health Organization is guiding LMIC to develop a health financing system to achieve and maintain UHC, where the national HI system has been promoted as a vital health financing strategy to expand pooled funds for equitable financing of health care [2].

Vietnam has achieved remarkable results in health care, reflected in some basic health indicators: the average life expectancy is 73, infant mortality rate per 1,000 live births is 14.7, maternal mortality rate per 1,000 live births is 54, malnutrition rate of children under the age of 5 is 14.1%, proportion of fully vaccinated children is 90%, and the share of population with access to improved sanitation facilities is 75% [6, 7]. However, in recent years, there has been an increase in inequity in health between different regions and ethnic and income groups [8]. For example, the percentage of children who had an episode of diarrhoea among ethnic minority households is 2.8 times higher than that among Kinh/Hoa households. The corresponding figures for the lowest and highest quintile households are 5.2% and 15.4%, respectively [9]. Inequality in human resources for health also exists. The number of doctors per 10,000 people in the capital city is 9, but in remote areas, this figure is 1 [10]. The number of doctors at the grassroots level is 1,995, while at the provincial level, it is 5,304. The numbers of nurses with high degrees at these facilities are 140 and 1,920, respectively. The percentage of commune health stations with doctors is only 78% [6].

**Figure 1:**
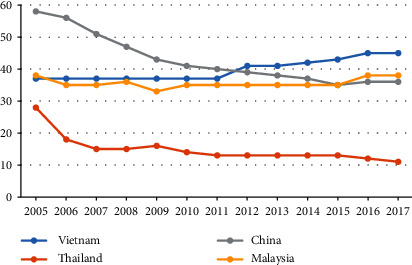
OOP as % of the current health expenditure in select Asia Pacific countries [11].

**Figure 2:**
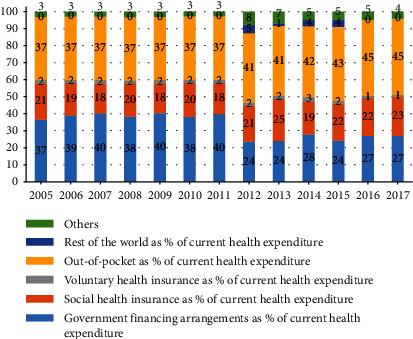
Structure of health financing resources, 2005–2017 [11].

The Vietnamese government has made significant efforts to scale up public financial resources allocated to health to achieve UHC. The current health expenditure accounted for 6% of the gross domestic product in 2017, which was similar to those of neighbouring countries and other LMIC, such as China (5%), Cambodia (6%), Myanmar (5%), and the Philippines (4%). The per capita public expenditure on health (in purchasing power parity international dollar) increased from 69 in 2005 to 183 in 2017 [11], surpassing the benchmark of 86 set by the World Health Organization (WHO) [12]. However, Vietnam has moved away from certain benchmarks to achieve UHC. For example, the share of government health expenditure in the gross domestic product has fluctuated around 3% during the last decade [11]. According to the WHO guidelines, if this share is less than 5%, the health system depends significantly on out-of-pocket health expenditure (OOP) [12, 13]. The proportion of OOP in the current health expenditure in Vietnam remains higher than those of Thailand, China, and Malaysia [11] (Figure 1). About 45% of the total health expenditure originates from OOP, while the benchmark proposed by the WHO is 15%–20%. This means that it is difficult to achieve the target of UHC when OOP constitutes more than 20% of the total health expenditure. The share of public health spending (state budget and social HI) experienced a significant decline from 58% in 2005 to 50% in 2017 (Figure 2). Additionally, government health expenditure as a share of total government spending remained stable at around 9% [11]. The share of OOPs in total health spending, therefore, increased substantially from 37% to 45% between 2005 and 2017.

The impact of HI on the utilisation of health care services and OOP in LMIC is demonstrated by several published studies. Most studies postulate a positive impact of HI on health care utilisation [14–22]. For example, using the difference-in-difference (DID) method and data from health utilisation and expenditure surveys, Gotsadze et al. find that medical insurance for the poor in Georgia increased the use of formal health services by 12% [16]. Similarly, applying the fixed-effects model with instrumental variables, Liu and Zhao show that subsidised voluntary public HI programmes in China increased outpatient care utilisation by 7%–13% and the number of hospitalised days by 0.35–0.5 days [18]. Erlangga et al. also investigate that Jaminan Kesehatan Nasional in Indonesia increased inpatient admission for the premium voluntarily paid group by 8.2% and subsidised group by 1.8% [21]. Similarly, in Vietnam, Nguyen finds that student and free HI programmes increased the number of health care visits by 12.4% and 66.1%, respectively [23]. Guindon also suggests that the Health Care Fund for the Poor in Vietnam contributed to an increase of 0.068 in the number of inpatient visits [22].

However, evidence on the effect of HI on OOP reduction is inconsistent [14, 15, 24–26]. Many recent studies have shown that HI lowers OOP [16, 26–28]. However, Liu and Zhao find that the Urban Resident Basic Medical Insurance in China does not reduce OOP [18]. Another study in China indicates that the elderly participating in social HI spend more on total OOP than those without HI [29]. Additionally, Erlangga finds that the public HI programme in Indonesia has no statistically significant effect on OOP [30]. In Vietnam, while Axelson et al. demonstrate a negative effect of Vietnam's Health Care Fund for the Poor on OOP [31], Wagstaff shows that the Health Care Fund does not decrease OOP [32]. Nguyen confirms that the Free Health Insurance Programme for children aged under six reduces OOP per visit, whereas voluntary student HI programmes do not [23]. These contradictory results can be explained by the fact that these studies are conducted in different health settings with different HI policies and periods.

After the Vietnamese government passed the Revised HI Law in 2014, little quantitative evaluation has been conducted on the impacts of HI programmes on health care utilisation and OOP. This study—among the first to demonstrate these cause-effect relationships—is expected to contribute to empirical evidence in the following aspects. First, we reveal empirical findings on the impact of HI on groups that pay a premium based on family. This family-based health insurance programme, also known as VHI, has been recently introduced in Vietnam and has not been assessed in previous studies. Second, one of the most important recent health financing reforms of the Vietnamese government has been the swap from supply-side to demand-side subsidies, which is reflected in increasing the state budget to pay HI premium for the poor, near-poor, and other disadvantaged groups [33]. Therefore, evaluating the impact of HI on heavily subsidised groups can also provide important empirical evidence for policymakers in Vietnam. Third, to date, in Vietnam, far too little attention has been paid to examining the impact of HI on health care utilisation and OOP at different levels of health care providers. Fourth, the study uses more recent nationwide Vietnam Household Living Standard Surveys (VHLSS) 2014 and 2016. Therefore, the findings from these nationally representative data can be generalised to the whole population. Furthermore, regarding the health financing system, Vietnam and several LMIC share the same characteristics [34]. Hence, studying the performance of the HI scheme in terms of increasing access to health care services and reducing OOP can offer valuable experience to LMIC of moving toward UHC. Thus, this study is aimed at evaluating the impact of the HI schemes on health care utilisation and OOP using panel data from VHLSS 2014 and VHLSS 2016.

**Figure 3:**
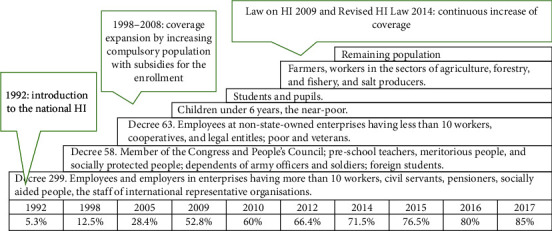
HI coverage expansion, 1992–2017 [34, 35].

*Overview of Vietnam's HI Programme*. The process of implementing universal HI in Vietnam has achieved considerable results with rapidly increasing HI coverage (Figure 3). The HI policy was first introduced in Vietnam in 1992, aiming at covering civil servants and employees in large- and medium-sized private enterprises. In 1993 (after one year of implementing the policy), the number of people covered by HI accounted for only 5.3% of the population; by 2017, this figure increased to approximately 85% [35] because, over the past 25 years, the government adopted several policies to expand HI coverage, removed financial barriers, and boosted access to health care services. In 2002, the government issued Decision 139, which established the Health Care Fund for the Poor [36]. In 2005, Decree 63 was adopted, which included some compulsory enrolment groups, such as workers in nonstate enterprises with less than 10 employees and workers in all organisations that are legally established and operating. Besides, under the decree, full subsidies for purchasing HI cards for the poor and ethnic minorities were provided. Consequently, the share of the population with HI increased sharply from 28.4% in 2005 to 42% in 2007 [35]. In 2009, the HI Law was enacted, forming a national/social HI scheme. According to the law, individuals formally employed, children under the age of six, elderly, poor, and near-poor were the compulsory HI-covered groups [36]. Some groups received heavy subsidies from the Vietnamese government, resulting in the enrolment rate increasing to about 60% in 2010 [36].

In 2014, the Vietnamese government enacted the Revised HI Law [37] and the National Assembly passed Decree 105/2014/ND-CP Guidance on implementing the HI Law [38], which stipulated the eligible groups of the population, premium contributions, subsidy levels from the state budget, determined copayment rates, and the participant's benefits. The law was officially effective from 1 January 2015. Vietnam Social Security is responsible for managing HI funds. In principle, Vietnam's HI scheme applies a single payer with a single financing pool and integrated benefits package [36]. According to the Revised HI Law, the HI scheme has been classified into five groups, in which, family-based contributions to HI premium have been added as group 5 [39]. Enrolment in HI is based on the individual and not the household level. This means that within a household, members might join different HI programmes with diverse premiums and subsidy levels and be entitled to several copayment rates. According to the revised law, HI membership is compulsory in Vietnam [37]. However, the government has been facing challenges when monitoring and compelling informal sector workers to participate. Consequently, the HI scheme in Vietnam continues to be a blend of compulsory and voluntary programmes [40].

**Figure 4:**
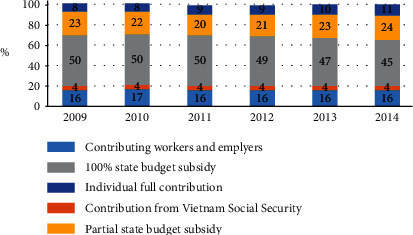
Trends and structure of HI coverage by entitlement groups, 2009–2014 [41].

In terms of HI coverage structure by entitlement groups, groups with full or partial subsidies from the government constituted the highest proportion of enrolees, accounting for 70% of the total enrolments (Figure 4) [41].

In terms of benefit packages, HI includes curative and preventive health care services: medical examination, treatment, functional rehabilitation, pregnancy checkups and delivery, screening, and early diagnosis of some diseases [37], except for primary health care services covered by national target programmes, such as vaccination, counselling, education and health promotion, surveillance and prevention of infectious diseases, and maternal and child health care [36, 40]. Health facilities covered by HI include the public and a small number of select private facilities having contracts with Vietnam Social Security [40]. The benefits packages were revised according to the HI Law 2014 and the entitlements extended for the enrolees, for example, covering costs of transporting patients from district hospitals to higher levels for some entitlement groups. Besides, the law now stipulates that the insured can visit any health facility at the district and commune levels without referring to letters [37, 40, 41]. The copayment rates were adjusted; for instance, the poor, ethnic minorities, policy beneficiaries (e.g., war veterans), people living in socioeconomically difficult areas, and islands are exempted from copayment and are entitled to free medical services; the copayment rate for the near-poor reduced from 20% to 5% [37, 39, 40]. With regard to provider payment methods, in Vietnam, there are three types of payment mechanisms: fee-for-service (the most popular method), capitation (applied mainly at district hospitals), and case-based or diagnostic-related groups (piloted in some provinces).

## 2. Methods

### 2.1. Data

We used data from the two recent rounds of VHLSS 2014 and 2016, which were carried out by the General Statistics Office of Vietnam and the World Bank. This is a large-scale national survey, representative of the whole country, rural and urban areas. While the 2014 survey covered 9,399 households and 35,920 individuals from 3,130 communes, the 2016 survey consisted of 35,793 individuals from 9,399 households, of whom 50% were the same households selected from the VHLSS 2014 and 50% were newly selected from the 2009 Census on Population and Housing of Vietnam. After matching two waves of VHLSS 2014 and VHLSS 2016, we obtained a balanced panel dataset comprising 30,180 observations from 15,090 individuals over 2 years.

The surveys included information on the types of HI cover of individuals, their health-seeking behaviour (i.e., the number of visits at different levels of health care providers—commune health centres, district hospitals, provincial hospitals, state and private health facilities, and traditional healers), and OOP reported in the last 12 months by interviewees. In addition, useful information on demographics and socioeconomic characteristics, such as age, gender, education, marital status, occupation status, household composition, expenditure and income, and region of residence, were included in the surveys.

### 2.2. Empirical Approach

The challenge in the empirical study is the need to create counterfactual evidence when addressing selection bias to evaluate the impact of a programme or intervention. As there may be a systematic heterogeneity between participants and nonparticipants with regard to their observed and unobserved characteristics, a direct outcome comparison among the insured and noninsured individuals can be biased. We applied the DID method and a combination of the propensity score matching (PSM) and DID methods to evaluate the impact of HI on OOP. The DID method assumes heterogeneity, which is not observed in the state of participation, but this factor is constant over time.

DID compares the differences in outcomes over the period with outcomes observed in the preintervention baseline survey between the treatment and control groups. Let us assume that there are two periods *t* = 0 and *t* = 1, where 0 implies a period before the programme is implemented and 1 indicates a period after the programme implementation. Let *T* be the treatment status, where *T*_1_ = 1 indicates individuals who are beneficiaries of the HI programme (i.e., the treatment group) at *t* = 1, whereas *T*_1_ = 0 denotes individuals not receiving entitlement of HI policy (i.e., the control group). Let *Y*_*t*_^T^ and *Y*_*t*_^C^ be the corresponding outcomes for the treatment and control groups in time *t*, respectively. Let *X* be a vector of observed characteristics of individuals and households. The DID method allows us to calculate the average impact of the programme as follows [22, 42]:

(1) $$\begin{array}{c} \text{DID}\left(X\right)=E\left(Y_{1}^{\text{T}}-Y_{0}^{\text{T}}\;|\;X,T_{1}=1\right)-E\left(Y_{1}^{\text{C}}-Y_{0}^{\text{C}}\;|\;X,T_{1}=0\right), \end{array}$$

The DID estimate can be derived from the regression model. Specifically, the estimating equation has the following form:

(2) $$\begin{array}{c} Y_{it}=\alpha +\beta T_{i1}+\gamma t+\delta \left(T_{i1}t\right)+\theta X_{it}+u_{i}+\varepsilon_{i}\;\left(t=0,1;i=1,\cdots ,n\right), \end{array}$$

where *α*, *β*, *γ*, *δ*, and *θ* are unknown parameters; *α* is a constant term; *β* captures the permanent differences in outcomes between the two groups; *γ* accounts for the combined effects of any time-variant unmeasured covariates (i.e., the group effect) but affects outcomes identically for the treatment and control groups (i.e., time trend common to both groups); *δ* is the coefficient on the interaction term reflecting the true effect of treatment (i.e., the average DID effect of the programme); *u*_*i*_ represents characteristics that do not change over time and are not observed; and *ε*_*i*_ is random error [42, 43].

The DID method is based on an important assumption—the parallel trend assumption. This means that without the programme, the trend of change in outcomes for both the treatment group (HI participants) and the control group (nonparticipants) is the same. In other words, the trend in the outcome among individuals without HI in the period after the Revised HI Law was introduced serves as a good counterfactual for what would have happened to the treatment group in the absence of treatment.

In observational studies, different econometric methods exist to control observed and unobserved characteristics that may influence participation in HI and potential outcomes. In this study, to estimate DID, we used ordinary least squares (OLS) regressions and fixed-effects regressions, where unobserved heterogeneity characteristics are controlled. Additionally, the combination of PSM and DID methods with panel data at individual and household levels was exploited, which allows us to reduce the risk of bias in the estimation. This combination can be found in the studies of Mebratie et al. [44], Balamiento [45], Gustafsson-Wright et al. [46], and Nguyen [14]. While unobserved confounders that are likely to affect the decision to participate in HI and outcomes of interest are not considered in the PSM method, DID and PSM combined with DID account for time-invariant unobserved confounders [47].

### 2.3. Definition of Variables

#### 2.3.1. Treatment and Control Groups

We have two treatment groups—the individuals covered by the VHI programme in 2016 and uninsured in 2014 and those covered by HSHI programmes (e.g., HI for the poor, near-poor, policy beneficiaries, and free HI card for some disadvantaged groups) in 2016 and had no HI in 2014. The control group includes individuals who had no HI in both 2014 and 2016. The number of observations in the VHI and HSHI treatment groups is 1,648 and 896, respectively. The number of observations in the control group is 4,770.

#### 2.3.2. Outcome Variables

These include probability of having outpatient visits and inpatient visits; the number of outpatient visits, inpatient visits, and total visits; the number of visits at the district hospital; the number of visits at the provincial hospital; probability of having outpatient OOP and inpatient OOP; OOP for outpatient care; inpatient admissions of individuals; and total OOP at different levels of health facilities for 12 months. OOP consists of spending on medication, treatment, checkups, consultation, diagnosis, medicines, and indirect medical expenditure, such as travelling, caring, accommodation, and allowances for physicians. We took the natural logarithm of the OOP outcome variables. We added one in OOP before taking the natural logarithm (ln(OOP + 1)) when individuals reported zero OOPs [3].

#### 2.3.3. Control Variables

These were the observed characteristics of individuals and households before and after introducing the Revised HI Law, which included age, gender, ethnicity, marital status, education level, occupation, household composition, access to clean water, toilet, expenditure and assets of household, reported health status of individuals, and geographical location. They are controlled to reduce selection bias. These variables were selected based on a review of several previous studies [14, 29, 31, 39, 44, 48–51]. The definitions of the variables used in the estimates are presented in Table 1.

## 3. Results

### 3.1. Descriptive Analysis

The descriptive statistics of patterns in utilisation of health services at different health facilities for the treatment and control groups of interest is provided in Table 2. The mean number of visits is reported in Table 2 for individuals who had outpatient and/or inpatient visits in the last 12 months. Overall, between 2014 and 2016, the probability of having outpatient and inpatient visits and the utilisation of outpatient and inpatient services for treatment groups increased, whereas the probability of having outpatient and inpatient visits among the control group was almost stable at approximately 30.4 and 3.7%, respectively. The number of outpatient visits among the control group also remained unchanged at approximately 2.2 times per person per year. Similarly, the use of health care services at the district hospitals increased significantly among the VHI and HSHI treatment groups. For instance, the number of visits at the district hospital among the insured of the HSHI treatment group increased from 0.451 times in 2014 to 0.763 times in 2016. While the enrolees of HSHI programmes visited the provincial hospitals more intensively, those of the VHI programme visited these health facilities less frequently. Besides, compared to inpatient care utilisation, the frequency of using outpatient health care was higher for both the insured and uninsured, ranging from 1.778 to 2.493 times per person in the last 12 months. The number of outpatient visits was the highest for the VHI group. The treatment groups tended to use inpatient services more intensively compared to the control group. For example, in 2016, the average number of inpatient admissions among the VHI treatment group was 0.355 times per year, while the figure for the control sample was only 0.152 times per year.

Table 3 presents descriptive statistics of OOP structure, including the probability of having outpatient and inpatient OOP, the means of OOP for outpatient visits, inpatient admissions, and total OOP in relation to the level of health providers for the treatment and control groups in the baseline year 2014 and follow-up year 2016. The means of OOP per person were quite high because we only calculated for individuals who used medical services in the past 12 months and had reported their health expenses.

The probability of having outpatient and inpatient OOP increased for both VHI and HSHI groups whereas the figures for the control group were stable at around 30% and 4%, respectively. Among the VHI enrolees, the average total OOP was VND 2,341.90 (approximately US$110.28) in 2014 and VND 3,059.87 (US$138.11) in 2016, which was considerably higher than that of the control group, with mean OOP of VND 1,852.11 (US$87.22) and VND 2,200 (US$99.29) in 2014 and 2016, respectively. Regarding the participants of HSHI programmes, the average total OOP was also higher compared to the control group and experienced an increase between 2014 and 2016 with VND 2,029.96 (US$95.59) and VND 2,328.69 (US$105.10), respectively.

Among the treatment groups, the VHI group had higher total OOP than the HSHI group in both years (VND 2,341.9 and 3,059.8 vs. VND 2,029.9 and 2,328.6). Increased OOP trends for outpatient visits were observed in both the treatment and control groups. In particular, the insured of the HSHI group experienced a pronounced increase from VND 928.07 (US$43.70) to VND 1,326.01 (US$59.85) between 2014 and 2016. While the OOP for inpatient admissions was significantly reduced for the HSHI groups from VND 7,399.82 (US$348.47) in 2014 to VND 5,566.98 (US$251.26) in 2016, this trend was not visible among the participants of the VHI.

Table 3 also provides information on the average OOP per visit at different levels of health care providers for the treatment and control samples. Generally, patients spent much more when using health services at a higher level of providers. The average OOP per visit at the district hospital of the comparison groups ranged from VND 423.69 to VND 1,254.97 (US$19.95 to US$56.64), while OOP per visit at the provincial hospital was considerably higher, between VND 3,348.49 (US$157.69) and VND 5,605.29 (US$252.99). The OOP per visit at provincial hospitals significantly decreased for both the VHI and HSHI groups, while it considerably increased for the control group. Also, the OOP per visit at district hospitals sharply decreased for the HSHI sample.

Several observable and unobservable variables can influence the differences in OOP among the enrolees and nonenrolees.  Table 4 provides summary statistics of the characteristics of the insured and noninsured before and after the Revised HI Law was introduced. Compared to nonenrolees, those covered by VHI were as follows: generally older, less likely to be males, unskilled workers, more likely to live in households with a high proportion of people above 60 years, have access to clean water and toilet, live in households with higher expenditure, and possess more assets, such as motorcycles, telephones, radios, televisions, and computers. Further, they were more likely to be ill a greater number of times and for a higher number of days in the last 12 months.

Table 4 also represents descriptive statistics of the observable characteristics of the HSHI group. Overall, compared to individuals without HI, participants of HSHI programmes were more likely to be elderly, ethnic minorities, less educated, unskilled workers, and live in households with a higher share of the elderly and less likely to have access to toilet. Further, they were more likely to be the poorest, have fewer assets, such as motorcycles, telephones, radios, televisions, and computers, a lower health status, and live in rural areas.

### 3.2. Impact of HI on the Utilisation of Health Care Services

Table 5 reports the estimates of the impact of the VHI and HSHI programmes on probability of having visits and changes in individuals' utilisation of health services. DID and combination of PSM and DID (PSM-DID) methods with panel data of 2014 and 2016 were applied for impact assessment. Overall, the HI programmes had statistically positive impacts on the utilisation of health care services.

The results of the DID method show that enrolment in the VHI and HSHI programmes increased the probability of having outpatient visits by around 5%. The VHI programme also increased the probability of seeking inpatient care among the insured by 2%. In addition, participation in the VHI and HSHI programmes was significantly associated with an increase of about 0.178–0.286 in the mean number of total visits per person per year. The magnitude of the impact of the VHI programme was larger (0.284 vs. 0.178 under pooled OLS estimates). The study also examines the impact of HI on the use of outpatient and inpatient health care services separately. We found that the VHI and HSHI programmes significantly increased the mean number of outpatient visits by 0.172 and 0.293 in the last 12 months, respectively. However, the HI scheme had no statistically significant impact on the mean number of inpatient admissions. The average treatment effect also shows that an increase between 0.339 and 0.342 in the mean number of visits at district hospitals was due to VHI enrolment, whereas the average treatment effect of HSHI programmes on the mean number of visits at district hospitals was lower, at about 0.258 annually. In addition, the results suggest that enrolling in the VHI programme led to an increase of approximately 0.167–0.179 in the mean number of visits at provincial hospitals. However, the results show that HI did not have any statistically significant impact on the number of visits at the provincial hospital for those participating in the HSHI scheme. When comparing the treatment groups who sought care at district and provincial hospitals, the results indicate that the size of impact for the former was larger than that of the latter.

Consistent with DID estimates, the PSM-DID estimates also demonstrate a positive impact of the VHI and HSHI programmes on the probability of seeking outpatient and inpatient care and utilisation of health care services. In comparison with DID, the estimates of the effect of HSHI on the number of outpatient visits and the total number of visits obtained using PSM-DID were higher (0.241 vs. 0.172 and 0.217 vs. 0.178, respectively). By contrast, the estimates of the impact of HSHI on the number of visits at the district hospitals under the PSM-DID method were smaller compared to the DID method (Table 5).

### 3.3. Impact of HI on OOP

Estimates of the impact of HI on OOP indicators of household members, including probability of having OOP, OOPs for outpatient visits, inpatient admissions, and at different health facilities are presented in Table 6. Across different treatment groups, the estimates demonstrated significant reductions in the OOP on outpatient care and total OOP, although the size of the impact of HI on the VHI group was larger than that on the HSHI group. Because we used a semilogarithmic regression equation for estimating the impact of HI reform on OOPs, this impact was calculated as follows: *e*^*β*^ − 1 [3].

Under the DID method, the results indicated that compared to the control group, the VHI group had approximately 3.8% and 1.4% higher probabilities of having outpatient and inpatient OOP, respectively. Similarly, HSHI programmes increased the probability of having outpatient OOP by 3.7%. However, the HSHI scheme was not found to impact the probability of having inpatient OOP. On the whole, HI had a statistically significant negative impact on outpatient OOP. The estimated results of the pooled OLS and fixed-effects specification were quite similar. The impact was more pronounced for the HSHI group. The policy lowered outpatient OOP for individuals participating in HSHI programmes by 34.2% ((*e*^−0.418^ − 1) × 100) compared with 25.2% ((*e*^−0.291^ − 1) × 100) for enrolees of VHI (columns 2 and 4 of Table 6). Similarly, the HI decreased OOP for participants of the VHI and HSHI programmes when they sought inpatient care services. Nevertheless, a statistically significant impact was only found for the VHI group. Inpatient OOP for the participants of VHI decreased by 41.4% and 43.2% under the pooled OLS and fixed-effects specifications, respectively. Overall, the HI scheme reduced the total OOP for the participants of VHI and HSHI, ranging from 19.8% to 30.8%. In terms of the health provider's level, we found that the HI considerably reduced OOP for the insured of the VHI and HSHI groups when they used health services at the district hospitals. The percentages of reduction were 72.3% for the former and 63.2% for the latter under the fixed-effects estimate. With regard to visiting provincial hospitals, while the HI scheme contributed to decreases of 32.1% in OOPs for the VHI group (column 1 of Table 6), the impact of HI was not statistically significant for the HSHI group. Among the insured of the VHI programme, the HI reform lowered OOP more for those using health care services at district hospitals than those visiting provincial hospitals with 70.9% and 32.1%, respectively (the last two rows of Table 6).

In the PSM-DID method, the patterns of the impact of HI on OOPs were generally consistent with the DID method. Nevertheless, the impact of HSHI on the probability of having outpatient OOP was quite higher under the PSM-DID method than under the DID method. Compared to the estimation results of DID, using the PSM-DID method yielded a higher impact of VHI and a lower impact of HSHI programmes on OOP for outpatient care. Under the DID estimates, the HI policy contributed to the reduction of 25.2% and 34.2% in outpatient OOP for the VHI and HSHI groups, respectively. However, the figures were 29.0% and 25.5% when applying the PSM-DID method (columns 2, 4, 6, and 8 of Table 6). Similarly, although HI policy's negative impacts on OOP for inpatient admissions were observed for both the VHI and HSHI groups, the statistically significant effect was only found in the VHI sample. Similar to results of the DID method, the HI scheme reduced OOP per visit at provincial hospitals by as much as 31.7% for the participants of VHI (column 5 of Table 4), and the HI policy did not statistically affect OOP of HSHI enrolees when they sought care at provincial hospitals.

## 4. Discussion and Conclusion

This study evaluates the impact of the HI programmes on health care utilisation and OOP in Vietnam using panel data from VHLSS 2014 and VHLSS 2016. To solve the problem of self-selection when citizens participate in HI and control for unobserved confounders, we used the DID method to determine the causal impact of the HI programme. Our major results indicate that the VHI and HSHI programmes increased the incidence of outpatient and inpatient visits and the utilisation of medical services and reduced OOP among the insured. The results indicate that HI significantly increased the probability of individuals having outpatient care and the utilisation of outpatient care and lowered OOP for outpatient care for both the VHI and HSHI groups. This effect remained the same under different specifications. Changes in HI policy can explain the increase in outpatient care utilisation and reduction in OOP; for example, cost sharing for the poor decreased from 5% to 0%, and that for the near-poor reduced from 20% to 5%. Besides, the insured do not have to incur copayment when they receive any medical examination and treatment at the commune level or when the cost per visit is lower than 15% of the basic salary [37, 40]. This finding broadly supports the work of other studies in evaluating the impact of HI on health care utilisation. Most studies demonstrate that HI significantly increased the number of health care visits [14, 16, 18, 21]. For example, it has been shown by Nguyen that participation in VHI results in a significant increase in the average number of outpatient visits by approximately 0.9137 [14]. In terms of reducing OOP, our estimation result is consistent with that of Sepehri et al. [3]. Using fixed-effects and random-effects models, they show that HI in Vietnam reduced OOP by 24% for VHI participants and 15% for participants of HI for the poor. These findings were also reported by Wagstaff [15]. Using the DID method, the author found that Vietnam's Health Care Fund for the Poor considerably reduced OOP. Additionally, Aji et al. found that HI for the poor and vulnerable groups (*Askeskin*) in Indonesia lowered OOP by 34% [24]. However, the result is contrary to that of Nguyen, who found that there was no statistically significant impact of VHI on OOP [14]. A possible explanation for this difference is that Nguyen used the VHLSS 2004 and VHLSS 2006 datasets. After the Revised HI Law was introduced in 2014, there was a difference in the entitlements for VHI participants [40].

Furthermore, the impact of the HI programme on OOP for inpatient admission was negative, although the estimates were only statistically significant for the VHI group. The reduction in inpatient OOP and the increase in the number of inpatient visits for those participating in the VHI programme suggest that the HI scheme in Vietnam has improved. No evidence that the HSHI programmes had reduced inpatient OOP might be related to the fact that there was no statistically significant impact of the HSHI programmes on the probability of hospitalisation and the intensity of using inpatient care among the insured. Besides, two waves of the VHLSS (2014 and 2016) might be a short period for HI to impact inpatient OOP. Several reasons can also explain no reduction in OOP for hospital admission among the HSHI enrolees. No cap on copayment spending is a significant contributory factor for high OOP among inpatients. Quality of care in Vietnam has improved; therefore, patients might seek high-tech services and imported drug brands for treatment, subject to high OOP [36]. Besides, the fee-for-service payment method, which is commonly applied in hospitals in Vietnam, motivates health care providers to oversupply services [40, 52, 53]. Therefore, the Ministry of Health and Vietnam Social Security could flexibly apply payment methods. For example, capitation should be implemented for outpatient care and case-based payment for inpatient treatment at district hospitals; case-based payment methods can also be applied to inpatient treatment in all state hospitals except for high-tech facilities. Besides, strengthening control of drug and pharmaceutical prices, reducing the copayment for the use of generic drugs to change patients' preference for expensive drugs, and stimulating the consumption of generic drugs should be priorities. Promotion is also needed to eliminate prejudice against locally produced and generic drugs. Additionally, the limitation in understanding insurance entitlements can make inpatients pay more in copayment than they should [36]. Further research is needed to explain the reasons for the estimation results more fully.

This study also found that participation in HI increased the health care utilisation and reduced OOP of both the VHI and HSHI groups when they visited a district hospital. This study supports evidence from previous observations of [3]. In addition, the HI programmes had a higher impact on health care utilisation at the district level than at the provincial level. It may be that these participants benefitted from the free cost of examination and treatment. In fact, before the Revised HI was enacted, the insured received 70% of reimbursement for inpatient admission and paid full medical cost for outpatient care if they visited a district hospital without a referral. However, after the enactment of the Revised HI Law, the insured can skip referral and seek inpatient care at any district hospital without bearing additional copayment [37, 40].

With regard to the provincial hospital level of care, this study indicated that the VHI enrolment increased the number of visits. In addition, participation in VHI lowered OOP. However, the impact of the HI programme on health care utilisation and OOP in the HSHI group was not significant. A possible explanation for this result might be due to the following: medical and nonmedical payments; physical barriers; and quality, attitude, and behaviours of health care providers toward the insured of the HSHI group, which may prevent them from accessing this level of care [36, 54–56]. Another possible explanation for the insignificant impact is the way we defined OOP to include not only user fees but also informal payments, such as bonuses for physicians, spending on additional medicines, and equipment/supplies. However, HI reimbursed only user fees and list of drugs.

Thus, the government could develop a policy of full exemption of copayment for the near-poor to reduce their OOP. In fact, the threshold for dividing the poor and near-poor is not substantially different. In addition, policymakers should develop policies for free patient transportation from district health facilities to higher levels of care and free meals for disadvantaged groups when they visit provincial hospitals. To protect households from massive OOP, the government could also introduce a threshold copayment policy. This means that patients do not have to copay if their monthly payment has attained a particular threshold. Furthermore, the Ministry of Health could improve the quality of medical care by issuing practice certificates, professional ethics, quality accreditation, and clinical practice guidelines, addressing unreasonable use of medicine problems via guidance on medical care practices.

## 5. Limitations

There are some limitations to this study. First, there could be measurement errors in the VHLSS surveys used. Some questions were based on self-reporting by interviewees, such as the number of outpatient and inpatient visits, the number of visits at health facilities in the past 12 months, OOP for outpatient care, and inpatient admissions, which can lead to inevitable and differential recall biases. Second, the variables used in the econometric model mainly address the demand-side. The variables that address the supply-side and the external environment, such as the number of health facilities and health workers, availability of drugs in every commune or district, local budgets for health, epidemic diseases, local disease control, natural disasters, environmental pollution, and local socioeconomic conditions, should be investigated in future research. Although we used panel data and a fixed-effects method to eliminate time-invariant unobserved characteristics, the bias cannot be removed entirely. Third, due to time and budget constraints, we could not conduct in-depth interviews to assess the impact of qualitative factors, such as attitudes of health staff, the insured patients, and the noninsured's satisfaction with medical services on health care utilisation as well as service providers' perceptions, views of local authorities, and policymakers. Such primary data could have helped create deeper insights into stakeholders' views and reduce potential evaluation bias. We believe that future research will fill this gap. Finally, the interval after the revision of the law may have been short to evaluate its impact. Future research may address these issues by using longitudinal data.

## Figures and Tables

**Table 1:** Definition of variables in evaluating the impact of HI on out-of-pocket expenditure.

Variables	Description
Treatment status	
Participation in VHI	Whether the household member participates in VHI (equals 1 if yes/0 if he or she does not participate in any HI programme)
Participation in HSHI programmes	Whether the household member participates in the HI programme for the poor, near-poor, policy beneficiaries such as meritorious people (equals 1 if yes/0 if he or she does not participate in any HI programmes)

Outcome variables	
Probability of having outpatient visits	Probability of visiting any of the health facilities for outpatient care by an individual in the last 12 months
Probability of having inpatient visits	Probability of visiting any of the health facilities for inpatient care by an individual in the last 12 months
Number of outpatient visits	Number of outpatient visits by an individual at any of the health facilities in the last 12 months
Number of inpatient visits	Number of inpatient visits by an individual at any of the health facilities in the last 12 months
Total visits	Total number of visits in the last 12 months
Number of visits at the district hospital	Number of visits at district hospital by an individual in the last 12 months
Number of visits at the provincial hospital	Number of visits at the provincial hospital by an individual in the last 12 months
Probability of having outpatient OOP	Probability of making outpatient OOP by an individual in the last 12 months
Probability of having inpatient OOP	Probability of making inpatient OOP by an individual in the last 12 months
Outpatient OOP	OOP for outpatient care in the last 12 months
Inpatient OOP	OOP for inpatient admission in the last 12 months
Total OOP	OOP for outpatient and inpatient care in the last 12 months
OOP per visit at the district hospital	OOP per visit at the district hospital in the last 12 months
OOP per visit at the provincial hospital	OOP per visit at the provincial hospital in the last 12 months

Explanatory variables	
Age group	Age of household members/individual (ordinal variable) equals
≤30	1 if the individual belongs to the age group equal or below 30
31–40	2 if the individual belongs to the age group 31–40
41–50	3 if the individual belongs to the age group 41–50
51–60	4 if the individual belongs to the age group 51–60
≥61	5 if the individual belongs to the age group equal to or above 60
Gender (male)	Gender of the individual (1 if male/0 if female)
Ethnicity (Kinh and Hoa)	Whether an individual belongs to ethnic Kinh/Hoa group (equals 1 if yes/0 if the individual belongs to a different ethnic minority group)
Marital status (married)	Marital status of the individual (1 if married/0 otherwise)
Education level	The education level of the individual (ordinal variable) equals
Not completed primary school	1 if the individual did not finish primary school
Primary school	2 if the individual completed primary school
Lower secondary	3 if the individual completed lower secondary school
Upper secondary	4 if the individual completed upper secondary school
Vocational school	5 if the individual completed vocational school
College, university, master, PhD	6 if the individual completed college, university, master, PhD
Occupation status	Occupation status of the individual (categorical variable) equals
Professionals/technicians	1 if the individual works as a professional or technician
Service or sales staff	2 if the individual works as a service or sales staff
Labourers in agriculture/forestry/fishery	3 if the individual works in agriculture or forestry or fishery
Manual labourers or machine operators	4 if the individual works as a manual labourer or machine operator
Unskilled workers	5 if the individual works as an unskilled worker
Others	6 if the individual does other jobs/or is not in the labour force
Household size	Total household members (continuous variable)
Household composition	
Share of children below 6 years	Share of children below 6 years in the household (continuous variable)
Share of the elders above 60 years	Share of the elders above 60 years in the household (continuous variable)
Access to clear water	Whether the household has access to clean water (1 if yes/0 if no)
Toilet access	Whether the household has access to a toilet (1 if yes/0 if no)
Expenditure quintiles	Based on household consumption expenditure data in the last 12 months, each individual in the household was ranked by their score. The ranking was then divided into five equal parts, from quintile one to quintile five. Each quintile group accounted for 20% of the sample. It is an ordinal variable and equals
First expenditure quintile group (poorest)	1 if the individual belongs to the first expenditure quintile (poorest)
Second expenditure quintile group	2 if the individual belongs to the second expenditure quintile
Third expenditure quintile group	3 if the individual belongs to the third expenditure quintile
Fourth expenditure quintile group	4 if the individual belongs to the fourth expenditure quintile
Fifth expenditure quintile group (richest)	5 if the individual belongs to the fifth expenditure quintile (richest)
Number of motorcycles	The number of motorcycles that household possesses (continuous variable)
Number of telephones	The number of telephones that household possesses (continuous variable)
Number of radio, television, or computer	The number of radio, television, or computer that household possesses (continuous variable)
Total residential area (m^2^)	The total residential area that household has (continuous variable)
Number of illness (times) in the last 12 months	Number of times that individual had an illness or severe injury in the last 12 months (continuous variable)
Number of illness (days) in the last 12 months	Number of days that individual had an illness or severe injury in the last 12 months (continuous variable)
Place of residence (urban)	Whether an individual lives in an urban area (1 if yes/0 if he or she lives in a rural area)
Region	The region where individual lives (categorical variable). It equals
Red River Delta	1 if the individual lives in the Red River Delta region
Northern Midlands and Mountains	2 if the individual lives in the Northern Midlands and Mountains
North and South Central Coast	3 if the individual lives in North and South Central Coast
Central Highlands	4 if the individual lives in Central Highlands
South East	5 if the individual lives in South East
Mekong River Delta	6 if the individual lives in Mekong River Delta

**Table 2:** Descriptive statistics of health care utilisation across different samples in 2014 and 2016.

Variables	VHI							
	2014				2016			
	Treated (*N* = 824)		Control (*N* = 2,385)		Treated (*N* = 824)		Control (*N* = 2,385)	
	Mean	SD	Mean	SD	Mean	SD	Mean	SD

Probability of having outpatient visits	0.320	0.467	0.304	0.460	0.434	0.495	0.308	0.461
Probability of having inpatient visits	0.049	0.217	0.037	0.188	0.109	0.312	0.039	0.195
Number of outpatient visits	2.422	3.519	2.218	2.714	2.493	3.637	2.312	2.336
Number of inpatient visits	0.158	0.418	0.133	0.407	0.355	1.030	0.152	0.542
Total visits	0.911	2.406	0.781	1.892	1.459	2.991	0.842	1.787
Number of visits at the district hospital	0.351	1.061	0.327	0.880	0.867	3.151	0.281	0.941
Number of visits at the provincial hospital	0.742	1.661	0.343	1.030	0.616	1.631	0.427	1.365

Variables	HSHI							
	2014				2016			
	Treated (*N* = 448)		Control (*N* = 2,385)		Treated (*N* = 448)		Control (*N* = 2,385)	
	Mean	SD	Mean	SD	Mean	SD	Mean	SD

Probability of having outpatient visits	0.310	0.463	0.304	0.460	0.408	0.492	0.308	0.461
Probability of having inpatient visits	0.060	0.238	0.037	0.188	0.095	0.294	0.039	0.195
Number of outpatient visits	1.778	1.800	2.218	2.714	2.439	2.715	2.312	2.336
Number of inpatient visits	0.185	0.435	0.133	0.407	0.275	0.687	0.152	0.542
Total visits	0.710	1.389	0.781	1.892	1.254	2.268	0.842	1.787
Number of visits at the district hospital	0.451	1.115	0.327	0.880	0.763	1.665	0.281	0.941
Number of visits at the provincial hospital	0.259	0.541	0.343	1.030	0.522	1.616	0.427	1.365

SD: standard deviation; *N*: number of observations.

**Table 3:** Descriptive statistics of OOP across different samples in 2014 and 2016.

Variables	VHI							
	2014				2016			
	Treated (*N* = 824)		Control (*N* = 2,385)		Treated (*N* = 824)		Control (*N* = 2,385)	
	Mean	SD	Mean	SD	Mean	SD	Mean	SD

Probability of having outpatient OOP	0.31	0.46	0.30	0.46	0.40	0.49	0.31	0.46
Probability of having inpatient OOP	0.05	0.22	0.04	0.19	0.10	0.30	0.04	0.19
Outpatient OOP	1186.15	2207.17	985.33	1865.84	1135.34	2682.71	1382.36	2965.20
Inpatient OOP	8984.15	15717.05	8539.96	22330.11	9831.26	24224.66	8178.68	15779.14
Total OOP	2341.90	6819.74	1852.11	8056.38	3059.87	11989.11	2200.01	6738.59
OOP per visit at the district hospital	423.69	425.99	804.38	1441.37	681.68	1479.83	776.63	973.65
OOP per visit at the provincial hospital	3729.94	8722.14	3348.49	9016.72	3374.31	5281.88	3991.41	8967.93

Variables	HSHI							
	2014				2016			
	Treated (*N* = 448)		Control (*N* = 2,385)		Treated (*N* = 448)		Control (*N* = 2,385)	
	Mean	SD	Mean	SD	Mean	SD	Mean	SD

Probability of having outpatient OOP	0.31	0.46	0.30	0.46	0.35	0.48	0.31	0.46
Probability of having inpatient OOP	0.06	0.24	0.04	0.19	0.09	0.29	0.04	0.19
Outpatient OOP	928.47	1316.48	985.33	1865.84	1326.01	4197.43	1382.36	2965.20
Inpatient OOP	7399.82	11255.55	8539.96	22330.11	5566.98	7514.78	8178.68	15779.14
Total OOP	2029.96	5283.36	1852.11	8056.38	2328.69	5828.10	2200.01	6738.59
OOP per visit at the district hospital	1254.97	2672.46	804.38	1441.37	673.78	1750.07	776.63	973.65
OOP per visit at the provincial hospital	5605.29	10117.52	3348.49	9016.72	3448.19	5830.04	3991.41	8967.93

SD: standard deviation; *N*: number of observations. Exchange rate in 2014: VND 21,235 = US$1 and in 2016: VND 22,156 = US$1.

**Table 4:** Descriptive statistics of control variables.

Variable	2014						2016					
	Control group		Treatment group (VHI)		Treatment group (HSHI)		Control group		Treatment group (VHI)		Treatment group (HSHI)	
	Mean	SD	Mean	SD	Mean	SD	Mean	SD	Mean	SD	Mean	SD
Age group												
≤30 (ref.)	0.04	0.22	0.02	0.21	0.05	0.23	0.43	0.47	0.41	0.46	0.41	0.47
31-40	0.22	0.41	0.17	0.38	0.18	0.38	0.16	0.36	0.11	0.31	0.11	0.31
41-50	0.34	0.47	0.35	0.48	0.32	0.47	0.18	0.39	0.16	0.36	0.15	0.36
51-60	0.25	0.44	0.28	0.45	0.25	0.43	0.13	0.33	0.18	0.39	0.15	0.36
≥61	0.15	0.36	0.18	0.38	0.20	0.40	0.10	0.30	0.14	0.34	0.18	0.38
Gender (male)	0.51	0.50	0.47	0.50	0.50	0.50	0.51	0.50	0.46	0.50	0.51	0.50
Ethnicity (Kinh, Hoa)	0.97	0.16	0.98	0.15	0.88	0.32	0.97	0.17	0.98	0.15	0.88	0.33
Marital status (married)	0.79	0.41	0.79	0.41	0.75	0.43	0.79	0.40	0.81	0.39	0.74	0.44

Education level												
Not completed primary school (ref.)	0.20	0.43	0.18	0.42	0.31	0.46	0.18	0.45	0.17	0.47	0.28	0.44
Primary school	0.30	0.46	0.28	0.45	0.28	0.45	0.31	0.46	0.29	0.45	0.30	0.46
Lower secondary	0.33	0.47	0.32	0.47	0.30	0.46	0.32	0.47	0.31	0.46	0.30	0.46
Upper secondary	0.11	0.32	0.14	0.35	0.08	0.27	0.11	0.31	0.13	0.34	0.06	0.24
Vocational school	0.05	0.22	0.06	0.24	0.03	0.16	0.06	0.23	0.08	0.27	0.05	0.21
College, university, master, PhD	0.01	0.12	0.02	0.13	0.00	0.07	0.02	0.13	0.02	0.15	0.01	0.11

Occupation status												
Professionals/technicians (ref.)	0.01	0.08	0.01	0.08	0.00	0.05	0.01	0.08	0.01	0.10	0.01	0.08
Service and sales staff	0.15	0.36	0.19	0.39	0.07	0.26	0.16	0.37	0.19	0.40	0.07	0.26
Labourers in agriculture/forestry/fishery	0.10	0.30	0.11	0.31	0.15	0.35	0.08	0.27	0.11	0.31	0.14	0.35
Manual labourers and machine operators	0.21	0.41	0.18	0.38	0.12	0.33	0.22	0.42	0.22	0.42	0.15	0.36
Unskilled workers	0.44	0.50	0.38	0.49	0.51	0.50	0.44	0.50	0.35	0.48	0.49	0.50
Others	0.10	0.30	0.14	0.35	0.15	0.36	0.09	0.28	0.12	0.32	0.14	0.35
Household size	4.25	1.37	4.24	1.48	4.33	1.67	4.22	1.44	4.24	1.55	4.21	1.80

Household composition												
Share of children below 6 years	0.08	0.13	0.07	0.11	0.08	0.13	0.08	0.12	0.07	0.12	0.08	0.12
Share of the elders above 60 years	0.09	0.18	0.11	0.21	0.15	0.28	0.10	0.20	0.13	0.23	0.17	0.30
Access to clear water	0.79	0.41	0.86	0.34	0.80	0.40	0.79	0.41	0.85	0.36	0.80	0.40
Toilet access	0.64	0.48	0.76	0.43	0.44	0.50	0.72	0.45	0.81	0.39	0.54	0.50

Expenditure quintiles												
First expenditure quintile group (poorest) (ref.)	0.22	0.42	0.15	0.40	0.32	0.45	0.16	0.36	0.09	0.35	0.23	0.42
Second expenditure quintile group	0.25	0.43	0.23	0.42	0.26	0.44	0.22	0.41	0.17	0.38	0.22	0.41
Third expenditure quintile group	0.21	0.41	0.26	0.44	0.16	0.37	0.24	0.43	0.22	0.42	0.22	0.41
Fourth expenditure quintile group	0.17	0.38	0.18	0.39	0.16	0.37	0.20	0.40	0.27	0.44	0.23	0.42
Fifth expenditure quintile group (richest)	0.15	0.35	0.18	0.39	0.10	0.30	0.18	0.38	0.25	0.44	0.10	0.30
Number of motorcycles	1.36	0.79	1.50	0.84	1.16	0.82	1.49	0.81	1.57	0.83	1.20	0.87
Number of telephones	1.75	1.02	1.90	1.07	1.49	1.02	1.92	1.10	2.07	1.14	1.58	0.96
Number of radio, television, or computer	1.18	0.52	1.29	0.64	1.06	0.43	1.21	0.59	1.34	0.65	1.09	0.48
Number of illness (times) in last 12 months	0.05	0.37	0.08	0.37	0.11	0.38	0.07	0.43	0.24	0.85	0.22	0.74
Number of illness (days) in last 12 months	0.67	9.51	1.17	7.33	2.00	9.88	0.74	5.30	3.18	13.08	2.00	7.14
Place of residence (urban)	0.26	0.44	0.26	0.44	0.14	0.34	0.26	0.44	0.26	0.44	0.14	0.34

Region												
Red River Delta (ref.)	0.26	0.42	0.26	0.43	0.17	0.36	0.26	0.44	0.26	0.43	0.17	0.38
Northern Midlands and Mountains	0.08	0.27	0.10	0.30	0.15	0.35	0.08	0.27	0.10	0.30	0.15	0.35
North and South Central Coast	0.20	0.40	0.25	0.43	0.38	0.48	0.20	0.40	0.25	0.43	0.38	0.48
Central Highlands	0.07	0.26	0.05	0.22	0.08	0.27	0.07	0.26	0.05	0.22	0.08	0.27
South East	0.12	0.33	0.12	0.33	0.05	0.23	0.12	0.33	0.12	0.33	0.05	0.23
Mekong River Delta	0.27	0.44	0.22	0.42	0.17	0.38	0.27	0.44	0.22	0.42	0.17	0.38

SD: standard deviation.

**Table 5:** Impact of HI on the utilisation of health care services.

Outcome variable	DID				DID with PSM			
	VHI		HSHI		VHI		HSHI	
	Pooled OLS	Fixed-effects	Pooled OLS	Fixed-effects	Pooled OLS	Fixed-effects	Pooled OLS	Fixed-effects
Probability of having outpatient visits	0.052^∗∗∗^ (0.013)	0.052^∗∗^ (0.018)	0.059^∗∗∗^ (0.017)	0.058^∗∗∗^ (0.011)	0.046^∗∗∗^ (0.014)	0.046^∗∗^ (0.015)	0.080^∗∗∗^ (0.0198)	0.079^∗∗∗^ (0.016)
Probability of having inpatient visits	0.019^∗∗∗^ (0.007)	0.020^∗∗∗^ (0.003)	0.009 (0.009)	0.010 (0.014)	0.016^∗∗^ (0.007)	0.017^∗∗∗^ (0.001)	0.004 (0.010)	0.003 (0.015)
Number of outpatient visits	0.293^∗^ (0.155)	0.300 (0.149)	0.166 (0.138)	0.172^∗∗^ (0.052)	0.288^∗^ (0.166)	0.287^∗∗^ (0.076)	0.226 (0.163)	0.241^∗^ (0.101)
Number of inpatient visits	0.030 (0.029)	0.033 (0.019)	-0.011 (0.032)	-0.008 (0.049)	0.010 (0.033)	0.016 (0.009)	-0.078 (0.043)	-0.079 (0.075)
Total visits	0.284^∗∗∗^ (0.072)	0.286^∗∗^ (0.089)	0.178^∗∗∗^ (0.066)	0.179 (0.090)	0.257^∗∗∗^ (0.076)	0.261^∗∗^ (0.071)	0.219^∗∗^ (0.073)	0.217^∗∗^ (0.072)
Number of visits at the district hospital	0.342^∗∗∗^ (0.100)	0.339^∗∗^ (0.113)	0.258^∗∗∗^ (0.072)	0.258^∗∗∗^ (0.041)	0.370^∗∗∗^ (0.105)	0.359^∗∗^ (0.092)	0.191^∗∗∗^ (0.074)	0.194^∗∗^ (0.061)
Number of visits at the provincial hospital	0.179^∗∗^ (0.076)	0.167^∗∗^ (0.046)	−0.045 (0.074)	−0.046 (0.036)	0.184^∗∗^ (0.081)	0.164^∗∗∗^ (0.012)	-0.073 (0.100)	−0.077 (0.071)

^∗∗∗^ *p* < 0.01; ^∗∗^*p* < 0.05; ^∗^*p* < 0.1. DID = difference-in-difference; PSM = propensity score matching; OLS = ordinary least squares. Notes: clustered-robust standard errors are reported in brackets of fixed-effects estimates, robust standard errors are reported in brackets of pooled OLS estimates. Standard errors are adjusted for clustering at the household level. Control variables include age, gender, ethnicity, marital status, education level, occupation, household size, household composition, expenditure and other assets of household (motorcycles, telephones, radio, TV, or computer), access to clean water, toilet, reported health status of individuals, and geographical location.

**Table 6:** Impact of HI on OOP.

Outcome variable	DID				DID with PSM			
	VHI		HSHI		VHI		HSHI	
	Pooled OLS	Fixed-effects	Pooled OLS	Fixed-effects	Pooled OLS	Fixed-effects	Pooled OLS	Fixed-effects
Probability of having outpatient OOP	0.038^∗∗∗^ (0.013)	0.038 (0.195)	0.037^∗∗^ (0.017)	0.037^∗∗∗^ (0.008)	0.029^∗∗^ (0.014)	0.029 (0.018)	0.060^∗∗∗^ (0.019)	0.059^∗∗^ (0.018)
Probability of having inpatient OOP	0.014^∗∗^ (0.006)	0.015^∗∗^ (0.004)	0.007 (0.009)	0.008 (0.014)	0.012^∗^ (0.007)	0.012^∗∗∗^ (0.002)	0.005 (0.010)	0.004 (0.016)
Log of outpatient OOP	−0.278^∗∗∗^ (0.092)	−0.291^∗∗∗^ (0.059)	−0.422^∗∗∗^ (0.126)	−0.418^∗∗∗^ (0.042)	−0.315^∗∗∗^ (0.098)	−0.343^∗∗∗^ (0.038)	−0.285^∗^ (0.149)	−0.295^∗^ (0.130)
Log of inpatient OOP	−0.535^∗∗^ (0.257)	−0.565^∗∗^ (0.177)	−0.282 (0.239)	−0.315 (0.286)	−0.643^∗∗^ (0.302)	−0.645^∗^ (0.248)	−0.059 (0.273)	−0.058 (0.167)
Log of total OOP	−0.221^∗∗^ (0.090)	−0.229^∗∗^ (0.051)	−0.368^∗∗∗^ (0.123)	−0.362^∗∗∗^ (0.037)	−0.293^∗∗∗^ (0.095)	−0.310^∗∗∗^ (0.024)	−0.299^∗∗^ (0.142)	−0.305 (0.158)
Log of OOP per visit at district hospital	−1.236^∗∗∗^ (0.186)	−1.282^∗∗∗^ (0.081)	−1.012^∗∗∗^ (0.207)	−0.999^∗∗∗^ (0.102)	−1.396^∗∗∗^ (0.206)	−1.438^∗∗∗^ (0.096)	−1.014^∗∗∗^ (0.306)	−1.004^∗^ (0.263)
Log of OOP per visit at provincial hospital	−0.388^∗∗∗^ (0.148)	−0.408 (0.269)	−0.147 (0.204)	−0.149 (0.255)	−0.381^∗∗^ (0.153)	−0.385 (0.238)	−0.107 (0.262)	−0.095 (0.230)

^∗∗∗^ *p* < 0.01; ^∗∗^*p* < 0.05; ^∗^*p* < 0.1. DID = difference-in-difference; PSM = propensity score matching; OLS = ordinary least squares. Notes: clustered-robust standard errors are reported in brackets of fixed-effects estimates, robust standard errors are reported in brackets of pooled OLS estimates. Standard errors are adjusted for clustering at the household level. Control variables include age, gender, ethnicity, marital status, education level, occupation, household size, household composition, expenditure and other assets of household (motorcycles, telephones, radio, TV, or computer), access to clean water, toilet, reported health status of individuals, and geographical location.

## Data Availability

Data are available on request through the corresponding author (nttthuong@tueba.edu.vn).
